# Comparative Analysis of Metabolic Variation in Eggplant Fruit of Different Varieties Reveals Metabolites Important for Quality Traits

**DOI:** 10.3390/foods12244383

**Published:** 2023-12-05

**Authors:** Xiaohui Zhou, Hesbon Ochieng Obel, Songyu Liu, Yan Yang, Jun Liu, Yong Zhuang

**Affiliations:** 1 Institute of Vegetable Crops, Jiangsu Academy of Agricultural Sciences, Nanjing 210014, China; xhzhou1984@sina.com (X.Z.); obel.hesbon@yahoo.com (H.O.O.); liusongyu1@126.com (S.L.); yzyangyan890618@163.com (Y.Y.); kehl_lau@foxmail.com (J.L.); 2 Laboratory for Horticultural Crop Genetic Improvement, Nanjing 210014, China

**Keywords:** eggplant, fruit quality, primary metabolites, phenolic acids

## Abstract

Eggplant is one of the most important vegetable crops worldwide and has been considered to have great antioxidant activity. However, little information is available about the primary metabolic composition of the nutritional values of eggplant. Using a widely targeted metabolome approach, the current study investigated primary metabolic variation in 13 eggplant varieties with different morphologies. A total of 503 primary metabolites (amino acids, lipids, nucleotides, organic acids, vitamin, saccharides, and alcohols) and 170 phenolic acids were detected, among which 211 metabolites were differently accumulated. Metabolic pathway analysis of the differential metabolites revealed the significant enrichment of phenylpropanoid biosynthesis, arginine biosynthesis, alpha-linolenic acid metabolism, and linoleic acid metabolism. The higher levels of amino acids and lipids were related to the umami, soft, and waxy taste of eggplant fruit. The present work substantially contributes to the knowledge of primary metabolite compositions regarding fruit-eating quality and provides useful information for the future breeding of eggplant.

## 1. Introduction

Eggplant (*Solanum melongena* L.) belongs to the Solanaceae family and is an economically important vegetable crop worldwide [1]. It is native to Southeastern Asia, with a great proportion of world production highly concentrated in Asia and the Mediterranean basin, and China has the highest production and consumption in the world [2,3]. Eggplant fruits are nutritious, containing carbohydrates, proteins, vitamins, minerals, as well as bioactive compounds [4]. Eggplant fruits have been described as a good source of antioxidants for being rich in phenolic compounds, which exhibit high antioxidant properties and are beneficial for human health [5].

The evaluation of secondary metabolites, especially phenolic compounds in eggplant fruit, has been extensively studied [5,6,7,8,9]. The predominant compound of phenolic acids is 5-caffeoylquinic acid, also known as chlorogenic acid, which is considered to be the main contributor to eggplant antioxidant capacity [3,10]. However, certain primary metabolites contributing to nutritional value and eating quality have been mostly overlooked [5]. Primary metabolites, including sugars, vitamins, amino acids, organic acids, lipids, and nucleotides, are essential metabolites in fruits, which play an indispensable role in plant development, fruit taste, and aroma [11,12]. Limited research has been conducted to provide a comprehensive overview of the primary metabolite composition of eggplant fruit. Furthermore, the key components that contribute to fruit taste in different types of eggplant varieties are largely unknown.

Eggplant has diverse fruit morphology, including color, size, and shape, as well as different fruit taste among different varieties. The production and consumption of eggplant has strong regional characteristics, especially in China. Although some studies have reported the nutrients composition of eggplant fruit, little information is available about the comparison of the metabolite composition differences among eggplant varieties from different production/consumption areas in China. Widely targeted metabolomics have been applied in plant metabolite analysis in many species due to the advantages of high-throughput and sensitivity in effectively determining the nutrient composition [13]. Therefore, in the present study, the composition differences in primary metabolites along with phenolic acids in the fruit pulp of 13 eggplant varieties with different fruit morphologies were investigated by a widely targeted metabolome approach. The metabolite characteristics of the varieties were also explored through a weight gene co-expression network analysis (WGCNA). These findings will not only give a better understanding of metabolites composition in different types of eggplant fruits but also provide valuable information for the future breeding of eggplant.

## 2. Materials and Methods

### 2.1. Plant Material

A total of 13 representative varieties of eggplant with different fruit morphologies comprising different market classes were used in this study, including E1 (cv. Suqie 6), E2 (cv. Suqie 301), E3 (cv. Bulita), E4 (cv. Tewangda), E5 (cv. Suqie 801), E6 (cv. Qinfeng 2), E7 (cv. Suqie 11), E8 (cv. Suqie 13), E9 (cv. Suqie 9), E10 (cv. Hangqie 1), E12 (cv. Liyancainuo), E13 (cv. Suqie501), and E14 (cv. Heidabang). The varieties used in this study were consumed by people for years. The good or poor taste of these varieties were generally acknowledged by consumers, especially the local types E10 and E12 that have an excellent taste, and E3 and E4 that have a poor taste.

The plants were grown in a greenhouse at Luhe experimental station of Jiangsu Academy of Agricultural Sciences, China. Each variety contained three biological replicates; each biological replicate contained 5 fruits derived from 5 individual plants (one fruit per plant). The fruits were selected for their uniform size and no defects. All the fruits were harvested at the commercial maturity stage and were then washed and peeled. The fruit flesh (without peel) samples were immediately frozen in liquid nitrogen and stored at −80 °C for subsequent metabolite analysis.

### 2.2. Sample Preparation and Extraction

The sample preparation and metabolites identification and quantification were performed by widely targeted metabolomics at Wuhan MetWare Biotechnology Co., Ltd. (Wuhan, China, 114°28′38′′ E and 30°29′24′′ N) (www.metware.cn, accessed on 22 June 2021). All of the samples were freeze-dried using a vacuum freeze-dryer (Scientz-100F, Scientz, Zhejiang, China, 121°39′34′′ E and 29°52′30′′ N), and then the freeze-dried samples were crushed in a mixer mill (MM400, Retsch, Nordrhein-Westfalen, Germany, 6°45′52′′ E and 51°14′8′′ N) with a zirconia bead for 1.5 min at 30 Hz. Next, 100 mg of lyophilized powder was dissolved in 1.2 mL of a 70% methanol solution, vortexed 30 s after every 30 min for a total of six times, and then stored at 4 °C overnight. Following centrifugation at 12,000 rpm for 10 min, the extracts were filtrated before UPLC-MS/MS analysis.

### 2.3. UPLC Conditions and ESI-Q TRAP-MS/MS

The sample extracts were analyzed using a UPLC-ESI-MS/MS system (UPLC, SHIMADZU, Kyoto, Japan, Nexera X2, www.shimadzu.com.cn/, accessed on 22 June 2021; MS, Applied Biosystems, Waltham, MA, USA, 4500 Q TRAP, www.appliedbiosystems.com.cn/, accessed on 22 June 2021). The analytical processes were performed by Wuhan MetWare Biotechnology Co., Ltd. (www.metware.cn, accessed on 22 June 2021).

### 2.4. Multivariate Data Analysis and Statistical Analysis

Normalized metabolite data from the 13 varieties were used to compare the metabolites. The results of the hierarchical clustering analysis (HCA) of the samples and metabolites were visually presented as heatmaps with dendrograms. HCA, principal component analysis (PCA), and orthogonal partial least-squares discriminant analysis (OPLS-DA) were processed through the Metware cloud platform (http://cloud.metware.cn, accessed on 28 July 2021). To identify metabolites that were significantly regulated between groups, the criteria of VIP ≥ 1 and absolute log_2_ (fold change) ≥ 1 were employed. Furthermore, the annotated metabolites were mapped to the Kyoto Encyclopedia of Genes and Genomes (KEGGs) pathway database (http://www.kegg.jp/kegg/pathway.html, accessed on 28 July 2021).

### 2.5. Metabolite Network Analysis

Weighted gene co-expression network analysis (WGCNA) was conducted on the metabolites that were detected in all samples using WGCNA R package [14] according to Ning et al. [13]. Eigen-metabolites were computed for each module and were used to investigate the association with metabolite characteristics of different varieties. The hub metabolites of the modules were selected based on the degree of metabolites according to Ning et al. [13].

## 3. Results

### 3.1. Phenotypic Characterization of Different Eggplant Varieties

**Figure 1:**
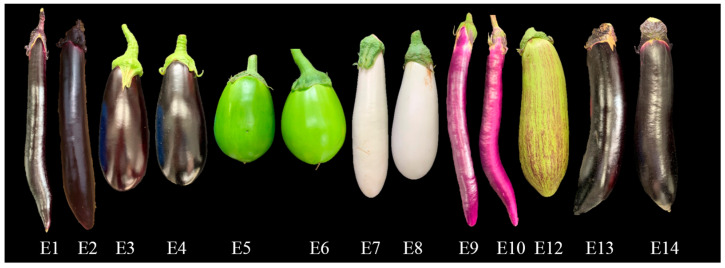
Fruit morphology of the thirteen eggplant varieties.

Eggplant fruits exhibit considerable phenotypic variability in fruit shape and color, such as ellipsoid, long shapes, and the fruit peel color being white, green, violet, purple, and black purple. The 13 eggplant cultivars used in this study were representative of the main variety types of the South Chinese market. The fruit morphologies of the 13 cultivars, particularly the fruit shape and peel color, were obviously different. They can be subdivided into seven market types, including E1 and E2 with dark purple fruit peel and purple calyx, E3 and E4 with dark purple fruit peel and green calyx, E5 and E6 with green fruit peel, E7 and E8 with white fruit peel, E9 and E10 with violet fruit peel, E12 with striped purple fruit peel, and E13 and E14 with dark purple fruit peel and a larger fruit diameter (Figure 1, Table 1).

The varieties used in this study have been consumed by people for years, and their good or poor taste are generally acknowledged by consumers. Typically, E3 and E4 belong to the European type, with firmer flesh and better storage life, but with poor taste. E1 and E2 were mostly consumed along the area of Yangtze River, which has medium firm flesh. E10 and E12 have strong regional characteristics. E10 is particularly cultivated and consumed mostly in Zhejiang Province of China, with better umami taste, while E12 is mostly cultivated and consumed in Southwestern China, with soft flesh and waxy taste.

### 3.2. Metabolomic Profiling of Fruit Flesh among Different Eggplant Varieties

To gain a comprehensive understanding of the primary metabolites and phenolic acids present in different eggplant varieties, a widely targeted metabolic profiling of fruit flesh was performed. Further, in order to ascertain the reliability of metabolites analyses, overlay analysis was performed for the assessment of the technical replicability of the metabolite detection. The TIC plots of the QC samples had a perfect overlap, implying that the detection of the metabolites of the same sample at different time points demonstrated excellent repeatability and instrumental stability (Figure S1).

**Figure 2:**
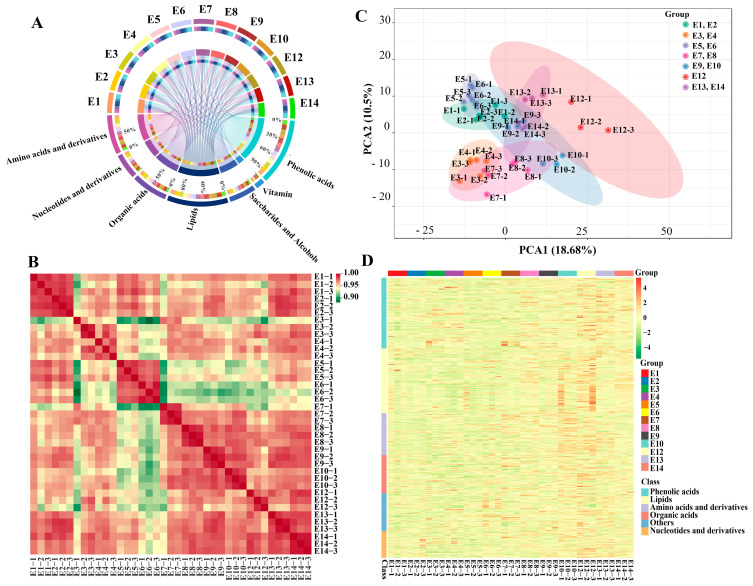
(**A**) Chord diagram, (**B**) correlation analysis, (**C**) PCA score plot, (**D**) and hierarchical clustering of the metabolites identified from the fruit flesh of the 13 eggplant varieties.

In total, 503 primary metabolites, including 100 amino acids and derivatives, 64 nucleotides and derivatives, 92 organic acids and derivatives, 69 saccharides and alcohols, 22 vitamins, and 156 lipids, were detected in this study (Figure 2A, Table S1). The 156 lipid compounds can be further attributed to six subclasses, i.e., 74 free fatty acids (FFAs), 32 lysophosphatidylcholines (LPCs), 27 LPEs, 17 glycerol esters, 3 sphingolipids, and 3 phosphatidylcholines (PCs). In addition to primary metabolites, a total of 170 phenolic acids were also identified among the 13 cultivars. The intragroup correlation analysis showed a high correlation between biological replicates (Figure 2B). Principle component analysis was carried out to compare the variation in the metabolite composition of the 13 eggplant cultivars. The cultivars belonging to the same market types were clustered well, except for E9 and E10. Although the 13 eggplant varieties had similar metabolic profiles, they can still be distinguished from each other (Figure 2C).

**Figure 3:**
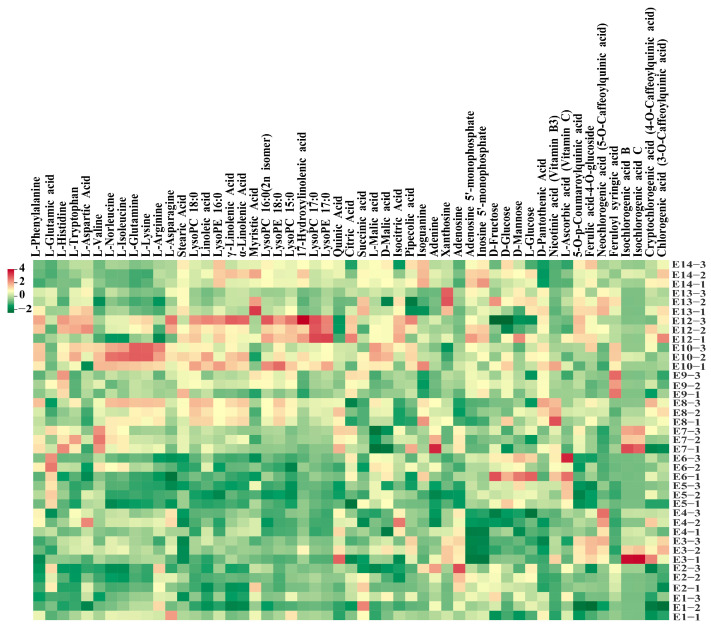
Heatmap of the main metabolites detected among the 13 eggplant cultivars.

Hierarchical cluster analysis (HCA) was also conducted to depict the metabolite variations among the 13 eggplant varieties (Figure 2D). In general, lipids, nucleotides and derivatives, and phenolic acids were more abundant in E12 with striped purple fruit peel, while most of the amino acids, including L-phenylalanine, L-tryptophan, L-isoleucine, L-lysine, L-glutamine, and L-arginine, were more abundant in E10. As for the sugar group, D-mannose, D-fructose, L-glucose, and D-glucose were present with a higher content in E5 and E6 (Figure 3).

### 3.3. Comparison of DAMs among Different Eggplant Varieties

Multiple comparisons using the criteria of VIP ≥ 1 and a *p* value < 0.05 was carried out to compare the metabolite composition differences in the 13 varieties. A total of 211 metabolites were differently accumulated. Among the 100 amino acids and derivatives detected for the 13 cultivars, a total of 32 were differently accumulated, including L-glutamic acid, L-aspartic acid, L-asparagine, and L-valine. Of the 156 detected lipids, fifty-four were differentially accumulated among all the samples, including lysoPE 16:0, lysoPC 15:0, lysoPC 17:0, myristic acid, and 17-hydroxylinolenic acid. Quinic acid, succinic acid, malic acid, and vitamin B3 were also differentially accumulated among the 13 cultivars. Detailed information of the DAMs among all the samples is illustrated in Supplementary Table S2.

**Figure 4:**
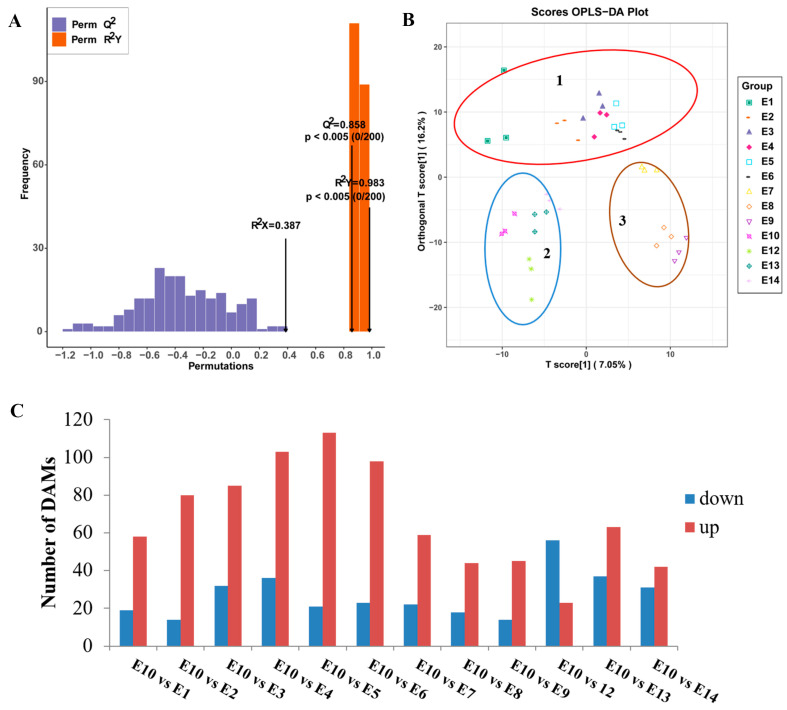
Orthogonal partial least-squares discriminant analysis (OPLS-DA) of multiple comparison among the 13 eggplant varieties. (**A**) OPLS-DA model of multiple comparisons. (**B**) OPLS-DA score plot of the 13 eggplant varieties. (**C**) Number of differentially accumulated metabolites among the 13 eggplant varieties.

In addition, the supervised method, OPLS-DA, was used to screen the variables responsible for differences in the multiple comparisons (Figure 4A,B). The Q2 value of the multiple comparison exceeded 0.85, demonstrating that the model was stable. The OPLS-DA score plots showed that the 13 varieties can be divided into three distinct groups: Group 1 included E1-E6; most of the metabolites had lower relative contents in these varieties. Group 2 included E10, E12, E13, and E14; most of the amino acids, lipids, and phenolic acids were highly accumulated in these four cultivars. Group 3 included E7, E8, and E9. Interestingly, although E9 and E10 had similar fruit peel color, the metabolites accumulation pattern was different.

**Figure 5:**
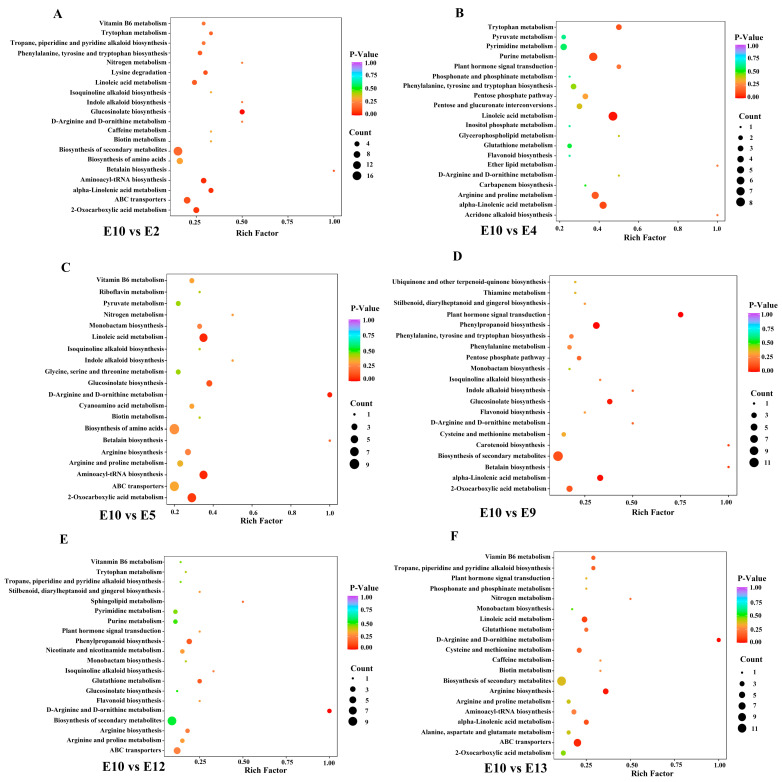
KEGGs enrichment analysis of the DAMs between E10 and E2 (**A**), E10 and E4 (**B**), E10 and E5 (**C**), E10 vs. E9 (**D**), E10 vs. E12 (**E**), E10 vs. E13 (**F**).

To further identify different accumulated metabolites (DAMs), the criteria of VIP ≥ 1 and fold change ≥ 2 or ≤0.5 were used between the comparison groups. As we focused on the exploration of the relationship between metabolites and fruit-eating quality, and most of the amino acids were highly accumulated in E10 based on the HCA analysis, E10, which had a better edible quality, was regarded as CK to compare with the other 12 varieties. The number of DAMs in different comparison groups (E10 vs. E1, E2, E3, E4, E5, E6, E7, E8, E9, E12, E13, and E14) was shown in Figure 4C. The Kyoto Encyclopedia of Genes and Genomes (KEGGs) enrichment analysis was also performed for the DAMs of each comparison group. The most enriched pathways detected for all comparison groups included phenylpropanoid biosynthesis, arginine biosynthesis, alpha-linolenic acid metabolism, and linoleic acid metabolism (Figure 5).

### 3.4. Identification of Metabolite Characteristics in Different Eggplant Varieties

**Figure 6:**
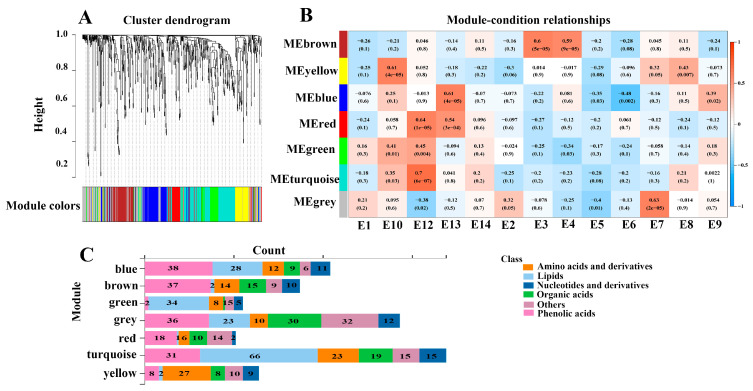
Correlation of metabolites with the eggplant fruit varieties based on WGCNA. (**A**) Clustering dendrogram for the identification of metabolite co-expression modules. (**B**) The associations between module and variety based on Pearson correlations. (**C**) Distribution of different types of metabolites in the detected modules.

To further explore the relation between metabolites and eggplant varieties with different phenotypes, WGCNA analysis was performed to correlate 673 metabolites with the eggplant varieties’ traits (Figure 6). Seven distinct modules which were labeled in different colors were obtained. The analyses revealed that the turquoise, yellow, blue, and grey modules had positive correlations with E12, E10, E13, and E7, respectively. In addition, the turquoise module was positively correlated with fruit mottling and the color of fruit mottling, but it was negatively correlated with fruit firmness, which was consistent with the typical characteristics of E12 (Figure S2). Likewise, E3 and E4 had firm fruit flesh, and the brown module was positively correlated with E3 and E4 varieties as well as fruit firmness.

The turquoise module contained 169 metabolites, which were more abundant in E12. In particular, 66 out of the 169 metabolites were lipids. Several LPEs and LPCs were more abundant in E12, which served as hub metabolites, including lysoPE 17:0, lysoPE 18:0, lysoPC 15:0 (2n isomer), lysoPC 18:0, lysoPC 16:0 (2n isomer), and lysoPC 17:0. The yellow module, which was correlated with the E10 variety, contained 64 metabolites, including 27 amino acids and derivatives, 9 nucleotides, 8 organic acids, 8 phenolic acids, 5 saccharides and alcohols, and 5 vitamins. The hub metabolites in the yellow module were mainly composed of amino acids, including L-isoleucine, L-glutamine, L-lysine, and L-norleucine. E10 and E12 varieties are rich in amino acids and lipids, suggesting that these metabolites may contribute to the better taste of E10 and E12.

## 4. Discussion

Although numerous studies on metabolites have been reported in eggplant, most of them focused on phenolic contents and their antioxidant capacity. Little information exists on the primary metabolite compositions of Chinese eggplant varieties, despite the fact that China is the largest area for eggplant production and consumption worldwide. Moreover, the nutritional components and contents, along with fruit taste quality, directly affect the commercial value of eggplant. In this respect, we focused on the exploration of primary metabolic composition variations among different Chinese eggplant varieties. The metabolites identified in this study will provide reference values for the selection and breeding of improved eggplant varieties with higher contents of nutritional and functional compounds.

### 4.1. Primary Metabolites Identified in the Fruit Flesh of Eggplant

In the present study, a total of 503 primary metabolites were detected in the 13 eggplant cultivars, including 100 amino acids and derivatives, 156 lipids, 64 nucleotides and derivatives, 92 organic acids and derivatives, 22 vitamins, and 69 saccharides and alcohols, indicating that the primary metabolites in eggplant fruit were rich and diverse. To the best of our knowledge, this is the first comprehensive overview of primary metabolite composition in Chinese varieties.

Amino acids and lipids are two important classes of nutrients and have various functions in plant physiology [15]. The most abundant amino acids detected in our study were phenylalanine, histidine, glutamic acid, tryptophan, and aspartic acid. However, glutamine, asparagine, and lysine were found to be the three most abundant amino acids in Turkish eggplant [16], while the main amino acids in Japanese eggplant were asparagine, glutamine, and arginine [17]. The differences in the detected amino acids between Chinese eggplant varieties and other types of eggplant varieties may be due to their different genetic background, as well as the different technology for metabolites detection. Lipids detected in the Chinese eggplant fruit were complex and mainly included free fatty acids, LPCs, LPEs, sphingolipids, and glycerol esters, among which stearic acid, lysoPC 18:0, linoleic acid, and linolenic acid were the most abundant, which was consistent with previous studies [8].

Sugars are important primary metabolites that possess nutritional value and contribute to the sweet taste of fruit [18]. In most fruits, glucose and fructose make up the majority of soluble sugars [19]. Previous studies on Turkish and Japanese eggplant fruit showed that the major soluble sugars were fructose, glucose, and sucrose, but in our study, mannose, glucose, and fructose were the predominant sugars.

Despite the fact that the composition of metabolites was similar for all 13 Chinese eggplant varieties, the concentrations of metabolites varied considerably. For instance, the relative contents of quinic acid were highly accumulated in E3 and E4, while the relative content of vitamin B3 was significantly higher in E8. Most of the lipids were more abundant in E12. In general, the samples belonging to the same commercial market type had a higher correlation. The local varieties seemed to have different metabolite accumulation patterns, demonstrating the potential of the landraces for the genetic improvement in metabolic traits.

Previous studies revealed correlations between plant metabolites and morphological characteristics of eggplant for both quantitative (fruit length, fruit diameter) and qualitative traits (fruit main color, fruit patches) [8], but in our study, morphology variation seemed to have no significant effect on the composition and contents variation in primary metabolites in eggplant fruit pulp. The different findings from the previous study [8] may be because of the different materials and methods as well as different metabolites used for correlation analysis. In the present study, the morphological traits, including fruit color, longitudinal diameter of fruit, transverse diameter of fruit, fruit calyx color, and fruit shape, showed a low correlation with flesh metabolites through WGCNA analysis (Figure S2). In addition, although the fruit peel color of E9 and E10 were both red–purple, the relative contents of the metabolites varied greatly, suggesting that it was more influenced by genetic diversity than by phenotypic variation that affects the accumulation of metabolites. Thus, metabolite accumulation in eggplant fruit is largely influenced by genetic factors, and geographical origin may also have an impact on metabolite composition.

### 4.2. Key Components Contributing to Fruit-Eating Quality of Eggplant

Fruit taste is an important aspect of the eating quality of eggplant, and primary metabolites are important contributors to fruit taste [11]. As the market demand for high-quality fruit is increasing, a more comprehensive understanding of fruit taste-related metabolites and their improvement is becoming an important objective in breeding programs [20]. The metabolome provides an essential means for the study of the nutrition and flavor of eggplant. Umami, soft, and waxy taste is an important index to measure the taste of eggplant fruit. In this study, the variation in the metabolite profiles of fruit pulp was possibly responsible for the differences in taste among different eggplant varieties. In particular, E10 and E12 seemed to have different metabolite accumulation patterns compared to other varieties based on OPLS-DA and WGCNA analysis, which is in accordance with their regional characteristics and better fruit taste.

The compositions and contents of amino acids are closely associated with fruit palatability, taste, and aroma [11,21]. Glutamic acid, aspartic acid, and lysine are the leading indicators for evaluating fruit taste [22,23]. Phenylalanine, tryptophan, and tyrosine are the precursors for aromatic compounds. Glycine and serine can give a sweet taste of fruit [24], whereas histidine, valine, leucine, isoleucine, and tryptophan are bitter amino acids [23,25]. Theanine and glutamic acid were found to be the key components that impart the umami taste of tea [12,25]. Apart from amino acids, 5′-nucleotides, including 5′-adenosine monophosphate (5′-AMP), 5′-ionsine monophosphate (5′-IMP), 5′-guanosine monophosphate (5′-GMP), and 5′-xanthosine monophosphate (5′-XMP), were also reported as umami compounds [22,26]. Considering that these 5′-nucleotides were not significantly differentially accumulated among all the varieties, and most of the amino acids were found to be more abundant in E10, including the umami-flavored components glutamic acid and aspartic acid, the better umami taste of E10 may be mainly attributed to highly accumulated amino acids. However, the 5′-nucleotides may work in synergy with amino acids to quantify umami intensity [22].

Previous studies have shown that lipid composition is an important regulator of fruit softness [27]. High levels of saturated fatty acids and linoleic acids, as well as a low index of unsaturated fatty acids, were correlated with fruit firmness in peaches [28]. Changes in fatty acyl chains that result in more rapid lysophospholipid accumulation are associated with date fruit ripening and softness [27], and glycerophospholipid metabolism was shown to be closely associated with the softening of pears at both the transcriptomic and metabolic level [27]. In apple, LPE18:1 was reported to be positively correlated to fruit firmness [29]. In addition, lipids are the main precursors of flavor substances and volatile organic compounds in fruit [23]. In our study, most of the lipids detected in our study showed high levels in E12, especially LPCs and LPEs. The relative contents of lysoPC 16:0 (2n isomer), lysoPC 18:0 (2n isomer), lysoPC 17:0, and lysoPE 16:0 (2n isomer) were significantly higher than those in E3 and E4, which had firm fruit flesh. E12 is a local eggplant variety with an especially soft and waxy fruit taste which is consumed mainly in Southwest China. Highly accumulated lipids may contribute to the soft and waxy taste of E12 fruit.

It is well known that the most important antioxidant compounds in eggplant are represented by chlorogenic acid (5-O-caffeoylquinic acid). In this study, 5-CQA, 5-O-p-coumaroylquinic acid, and ferulic acid-4-O-glucoside were the most abundant phenolic acids detected in Chinese eggplant varieties, which is similar to the findings of previous studies [9,30]. In our analysis, a large number of other less prevalent phenolic acids were also found. Apart from the health beneficial qualities of these phenolic acids, they were also reported to play key roles in fruit flavor and texture [31]. The highly accumulated phenolic acids in E12 were more likely to enrich the taste rather than bring an unpleasant taste. However, the composition and contents of metabolites have a complex influence on taste. The establishment of key metabolites in metabolic pathways will provide a theoretical basis for metabolomics-assisted breeding [32]. Future investigations will be necessary in order to comprehensively analyze the regulators of the metabolic pathways of key metabolites related to fruit taste, which in turn facilitate improvements in the eating quality of eggplant.

## 5. Conclusions

In this study, the composition differences in primary metabolites as well as phenolic acids in the fruit pulp of 13 Chinese eggplant varieties with different morphologies were investigated by a widely targeted metabolome approach. The composition and abundance of the metabolites varied among cultivars. A total of 503 primary metabolites and 170 phenolic acids were identified, and 211 metabolites were differently accumulated among the 13 varieties. Metabolic pathway analysis of the differential metabolites revealed the significant enrichment of phenylpropanoid biosynthesis, arginine biosynthesis, alpha-linolenic acid metabolism, and linoleic acid metabolism. The higher levels of amino acids and lipids were the primary contributors to better umami, soft, and waxy fruit taste for eggplant. Taken together, this study provides valuable information for the future breeding and sufficient utilization of eggplant.

## Figures and Tables

**Table 1:** Characteristics of the thirteen eggplant varieties.

Variety	Fruit Skin Color	Anthocyanin Coloration of Fruit Calyx	Fruit Flesh Color	Fruit Shape	Fruit Flesh Firmness
E1	Black purple	Present	Greenish	Cylindrical	Medium
E2	Black purple	Present	Greenish	Club shaped	Medium
E3	Black purple	Absent	Greenish	Ellipsoid	Strong
E4	Black purple	Absent	Greenish	Ellipsoid	Strong
E5	Green	Absent	Greenish	Obovate	Medium
E6	Green	Absent	Greenish	Obovate	Medium
E7	White	Absent	Whitish	Club shaped	Medium
E8	White	Absent	Whitish	Pear shaped	Medium
E9	violet	Present	Whitish	Cylindrical	Soft
E10	violet	Present	Whitish	Cylindrical	Soft
E12	Striped purple	Absent	Greenish	Ellipsoid	Soft
E13	Black purple	Present	Greenish	Club shaped	Soft
E14	Black purple	Present	Greenish	Club shaped	Soft

## Data Availability

The data supporting the results of this study are included in the present article.

## Supplementary Materials

The following supporting information can be downloaded at: https://www.mdpi.com/article/10.3390/foods12244383/s1, Figure S1: Total ions current overlaps of quality control samples by mass spectrometry detection. (A) TIC overlay plot in positive ionization mode. (B) TIC overlay plot in negative ionization mode; Figure S2: Module–trait relationships; Table S1: The primary metabolites and phenolic acids detected in the 13 eggplant varieties; Table S2: Detail information of the DAMs among all the 13 eggplant varieties.
