# Supplementary material for: Comparative Analysis of Metabolic Variation in Eggplant Fruit of Different Varieties Reveals Metabolites Important for Quality Traits
Source: Foods. 2023 Dec 5;12(24):4383. doi: 10.3390/foods12244383 (PMC10742729; doi:10.3390/foods12244383)
Supplement: Supplementary file 1 [file foods-12-04383-s001.zip › Supplementary Figures S1 and S2.pdf]

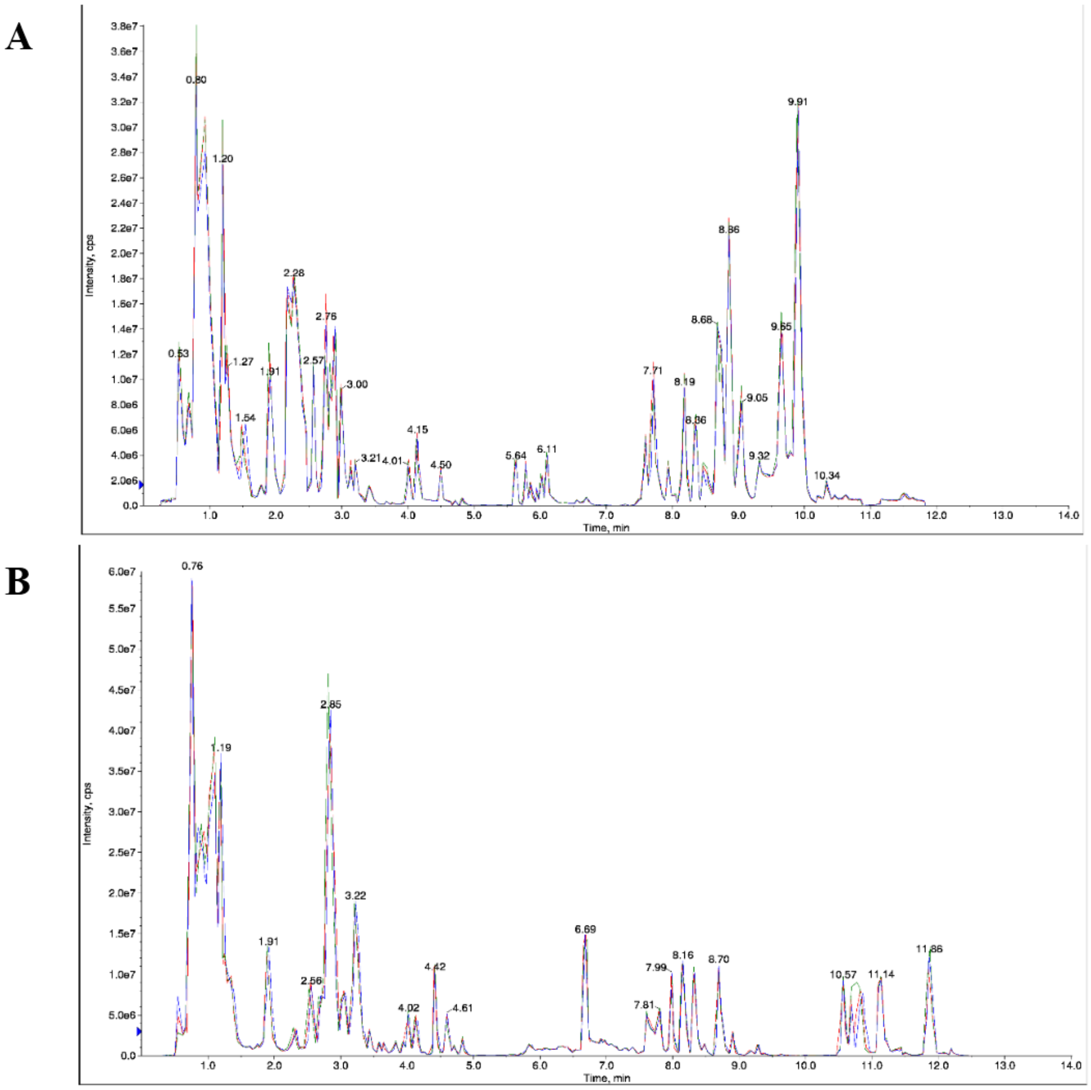

Supplementary Figure S1. Total ions current overlaps of quality control samples by mass spectrometry detection. （**A**） TIC overlay plot in positive ionization mode. （**B**） TIC overlay plot in negative ionization mode.

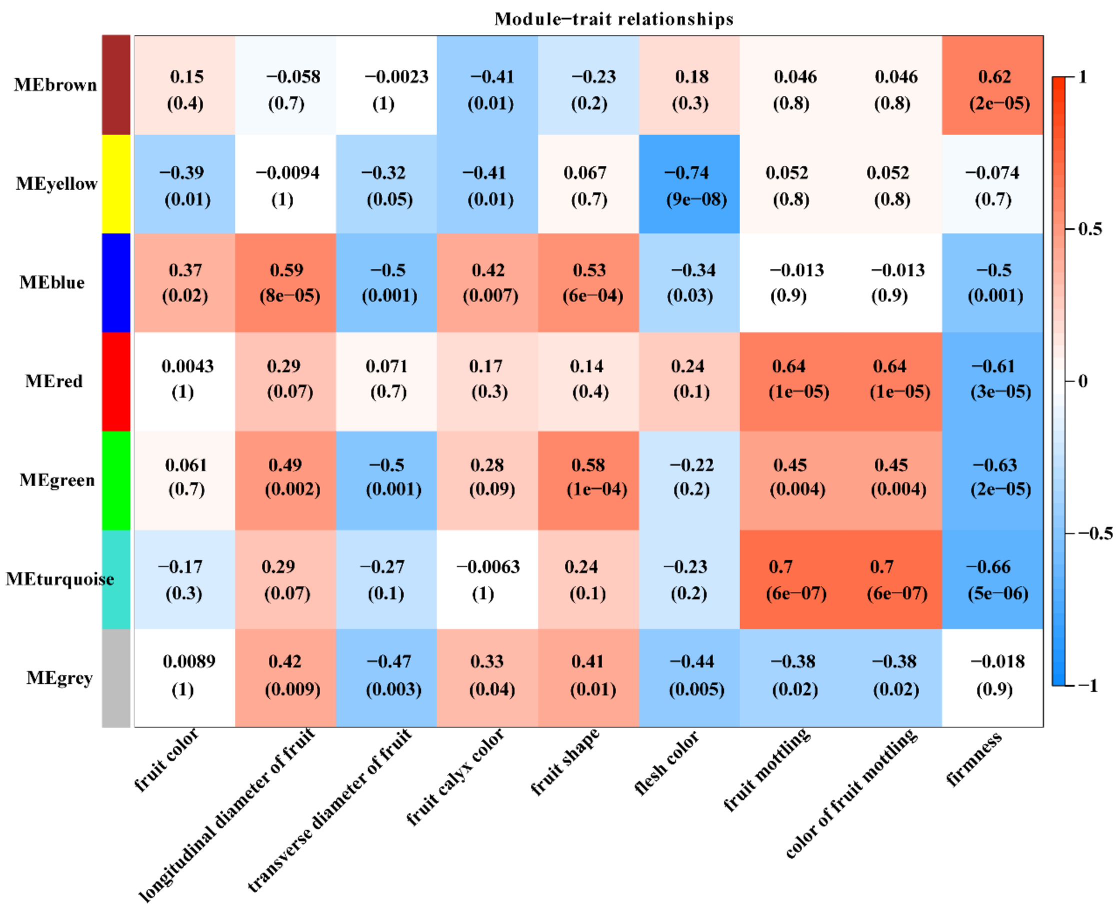

Supplementary Figure S2. Module-trait relationships.
